# Creating and Implementing a Principal Investigator Tool Kit for Enhancing Accrual to Late Phase Clinical Trials: Development and Usability Study

**DOI:** 10.2196/38514

**Published:** 2022-08-25

**Authors:** Kristin A Higgins, Alexandra Thomas, Nancy Soto, Rebecca Paulus, Thomas J George, Thomas B Julian, Sharon Hartson Stine, Merry Jennifer Markham, Maria Werner-Wasik

**Affiliations:** 1 Winship Cancer Institute Emory University Atlanta, GA United States; 2 Atrium Wake Forest Baptist Comprehensive Cancer Center Wake Forest University Winston-Salem, NC United States; 3 NRG Oncology Operations Center American College of Radiology Philadelphia, PA United States; 4 NRG Oncology Statistics and Data Management Center American College of Radiology Philadelphia, PA United States; 5 University of Florida College of Medicine Gainesville, FL United States; 6 Allegheny Health Network Cancer Institute Allegheny General Hospital Pittsburgh, PA United States; 7 NRG Operations Center American College of Radiology Philadelphia, PA United States; 8 Sidney Kimmel Cancer Center Thomas Jefferson University Philadelphia, PA United States

**Keywords:** clinical trial accrual, social media tools, principal investigator, PI toolkit, oncology, clinical trial, tool, resources, patient, investigators, accrual, development, engagement, study, community, planning, activation, social media

## Abstract

**Background:**

Accrual to oncology clinical trials remains a challenge, particularly during the COVID-19 pandemic. For late phase clinical trials funded by the National Cancer Institute, the development of these research protocols is a resource-intensive process; however, mechanisms to optimize patient accrual after trial activation are underdeveloped across the National Clinical Trial Network (NCTN). Low patient accrual can lead to the premature closure of clinical trials and can ultimately delay the availability of new, potentially life-saving therapies in oncology.

**Objective:**

The purpose of this study is to formally create an easily implemented tool kit of resources for investigators of oncology clinical trials within the NCTN, specifically the NRG Oncology cooperative group, in order to optimize patient accrual.

**Methods:**

NRG Oncology sought to formally develop a tool kit of resources to use at specific time points during the lifetime of NRG Oncology clinical trials. The tools are clearly described and involve the facilitation of engagement of the study principal investigator with the scientific and patient advocate community during the planning, activation, and accrual periods. Social media tools are also leveraged to enhance such engagement. The principal investigator (PI) tool kit was created in 2019 and thereafter piloted with the NRG Oncology/Alliance NRG-LU005 phase II or III trial in small-cell lung cancer. The PI tool kit was developed by the NRG Oncology Protocol Operations Management committee and was tested with the NRG/Alliance LU005 randomized trial within the NCTN.

**Results:**

NRG Oncology/Alliance NRG-LU005 has seen robust enrollment, currently 127% of the projected accrual. Importantly, many of the tool kit elements are already being used in ongoing NRG Oncology trials, with 56% of active NRG trials using at least one element of the PI tool kit and all in-development trials offered the resource. This underscores the feasibility and potential benefits of deploying the PI tool kit across all NRG Oncology trials moving forward.

**Conclusions:**

While clinical trial accrual can be challenging, the PI tool kit has been shown to augment accrual in a low-cost and easily implementable fashion. It could be widely and consistently deployed across the NCTN to improve accrual in oncology clinical trials.

**Trial Registration:**

ClinicalTrials.gov NCT03811002; https://clinicaltrials.gov/ct2/show/NCT03811002

## Introduction

Approximately 2% of all patients with cancer participate in a clinical trial in the United States [1]. The reason behind this seemingly low number is complex and multifactorial [2]—likely a combination of clinical trials with overly restrictive inclusion criteria, lack of access to clinical trials in some environments, low health literacy around clinical trials, and perhaps a generalized feeling of wanting to move forward with standard cancer-directed therapies.

In lung cancer, recent clinical trials have led to an explosion in US Food and Drug Administration approvals of new therapeutic agents in the last several years, including immunotherapies and targeted therapies. The American Cancer Society recently reported the highest percentage drop in cancer mortality, which was felt to be primarily related to improvements in lung cancer therapies [3]. Recent data also show that overall survival for non–small-cell lung cancer has significantly improved, which is in close correlation with the approval of new targeted therapies [4]. Taken together, these advances would not have been possible without the patients who enrolled in clinical trials investigating these therapies. However, there are significant barriers to patient recruitment and retention in oncology clinical trials, including restrictive eligibility criteria, financial barriers, logistical concerns, and uncertainty around experimental treatment arms that can make it difficult for patients to enroll [5,6]. Slow patient accrual to clinical trials ultimately impairs development of new therapies for patients.

The National Cancer Institute (NCI) National Clinical Trials Network (NCTN) is composed of 5 cooperative groups, which are as follows: The Alliance for Clinical Trials in Oncology, Children’s Oncology Group, The Eastern Cooperative Oncology Group-American College of Radiology Imaging Network (ECOG-ACRIN) cancer research group, NRG Oncology, and Southwest Oncology Group. It develops and conducts federally funded cancer trials across the United States and Canada [7,8]. Recent data evaluating the association of NCTN trials with guideline-based care and new drug indications found that nearly half of all phase-III Southwest Oncology Group trials were practice influential, meaning they either established the role of new cancer therapies or confirmed the benefits of standard of care therapies [9]. NCTN trials are critical to advancing cancer care, but many trials take years to complete accrual owing to lower-than-projected accrual rates.

To facilitate the goal of meeting projected clinical trial accrual, the NRG Oncology Protocol Operations Management (POM) Committee sought to develop, with input from the NRG Communications Committee, a tool kit for study principal investigators of newly activated NRG Oncology trials. Membership of these committees include physician leaders across various types of cancers who design and enroll patients on clinical trials. The physician membership includes a diverse array of people who practice in academia, the private sector, as well as underserved communities. Members also include patient advocates, statisticians, and administrative leaders with expertise in clinical trial design. The principal investigator (PI) tool kit was therefore developed with input from a wide array of stakeholders involved in clinical trial design, including patients themselves.

The goal of the PI tool kit is to harness communication-driven tools to message information about the trial stakeholders including patients, physicians, health care teams, advocacy groups, and oncology organizations. The tool kit works to create a patient-driven message, with a clinical trial patient advocate working with the principal investigator to cultivate a message that resonates with the patient community. This builds upon the concept of patient-centric clinical trials that involve patient input throughout the life cycle of the clinical trial [10]. Here, we describe the components of the PI tool kit, and present 18-month accrual data of the first NRG Oncology clinical trial to incorporate the PI tool kit: NRG/Alliance NRG-LU005 (registered with Clinical Trials.gov: NCT03811002). Importantly, the PI investigator tool kit is a newly created tool for clinical trial accrual, and this manuscript details a first pilot experience using the tool kit. Further studies using implementation science to integrate the PI tool kit into additional clinical trials are ongoing, albeit beyond the scope of this manuscript.

## Methods

### Tool Kit Development

In January 2019, NRG Oncology POM committee members, with input from the NRG Communications Committee, began developing the PI tool kit. It was developed based upon the cataloguing of best practices and was designed to be formulaic and easily broken down into discrete tasks to be performed at designated time points throughout the life cycle of the clinical trial. The tool kit tasks would be implemented by the overall study principal investigator with support from NRG Oncology operations staff. The study principal investigator is defined as the individual who has led the development of the clinical trial and who is responsible for the overall conduct of the clinical trial. The tool kit would use various awareness resources, communication tools, social media platforms (including Twitter), general oncology and disease-specific conferences, patient engagement websites, and disease-specific patient advocates. Patient advocates would partner with the study principal investigator to promote patient-centric messaging about the clinical trial and would be identified through the NRG Oncology patient advocacy committee.

The PI tool kit components as well as activation time points are detailed below. Of note, the PI tool kit focuses on methods to enhance patient recruitment and does not specifically address retention in clinical trials. Patient recruitment refers to the number of patients registered to participate in a clinical trial. The terms recruitment and accrual are used interchangeably. The PI tool kit is shown in Multimedia Appendix 1 and in the tables below.

### Ethical Considerations

The PI tool kit uses social media and scientific communications tools to enhance clinical trial accrual. The principal investigator tool kit was piloted using the clinical trial NRG Oncology/Alliance LU005, which is an approved clinical trial through the National Cancer Institute’s central institutional review board (CIRB; IRB00000781).

### Tools Used at Trial Activation

In the weeks to months leading up to study activation, a trial-specific Power Point slide deck is created that provides a succinct overview of the study, including the study rationale, patient population and inclusion or exclusion criteria, study schema, primary and secondary end points, expected accrual, and projected study length (Table 1). This is created by the protocol development team in the Operations and Statistics and Data Management Center offices, in collaboration with the study principal investigator. Upon completion and approval, it becomes available under “study documents” tab on the NCI’s Clinical Trials Support Unit (CTSU) website. A patient brochure is created for study sites to use as an educational tool for the trial. For select trials where resources are available, a study landing page is created on the NRG Oncology website for patients to easily engage with and obtain more information about the clinical trial and to identify possible involvement. The patient landing page and patient brochure development are assisted by the Communications Committee members. The patient landing website and brochure require CIRB approval and are available at both the NRG Oncology website and the CTSU protocol web page.

Upon study activation, an email communication is sent by the study principal investigators to the institutional NRG Oncology contact principal investigators (as well as to the institutional principal investigators for the study in question) to communicate that the new trial is activating and any specific information that may be of value to site principal investigators. The CIRB-approved patient brochure can also be sent with this email. The study principal investigator would also create a short 30- to 60-second video aimed at a patient audience, which describes the study patient population, study rationale, and study schema. This brief video script includes CIRB approval with the video initially shared on social media platforms such as Twitter by both the trial study principal investigator and NRG Oncology. Patient advocates are also encouraged to help develop this video to ensure the content is patient focused with clear messaging. NRG Oncology maintains an active Twitter account (“@NRGonc”) and Facebook page, both with a focus on engaging with the community and sharing information about cancer clinical trials. Lastly, the study principal investigator will conduct a study launch or kick-off session at the NRG semiannual meeting to educate clinical investigators and research staff on specific trial goals and requirements, as well as to stimulate overall study awareness.

**Table 1:** Principal Investigator (PI) tool kit—tools for study activation phase.

Tasks	Responsible party	Product or placement
Study overview slide set	Protocol Development or Communications Committee with Study Chair review	CTSU^a^ web page
Study landing page^b^ for patients	Protocol Development or Communications Committee with Study Chair review	NRG Oncology web page CTSU web page
Patient brochure	Protocol Development or Communications Committee with Study Chair review	NRG Oncology web page CTSU web page
Introductory letter to targeted sites with high accrual on similar trials or potential for high accrual	Protocol Development or Study Chair	Email from Study Chair
Patient-focused promotional video	Study Chair with or without patient advocate with support from Communications Committee	Study chair Tweets and NRG Oncology retweets
Study launch session	Protocol Development or Communications Committee with Study Chair review	NRG Oncology semiannual meeting

^a^CTSU: Clinical Trials Support Unit.

^b^Selected trials only.

### Tools Used During Trial Accrual Period

A second series of tools are used during the accrual period. Throughout the duration of the clinical trial, updates will be given by the study principal investigator at NRG Oncology semiannual meetings during disease-specific sessions that occur every 6 months (Table 2). Study NCTN champions will provide study updates at the different network group meetings on a semiannual basis. The study principal investigators will submit a “Trial in Progress” abstract to appropriate professional society meetings including the American Society of Clinical Oncology (ASCO) annual meetings and pertinent disease- and modality-specific society meetings. The purpose of “Trial in Progress” abstracts is to raise awareness about the trial among other health care professionals with the goal of increasing the number of study sites that have the trial open and available for patients. The study principal investigator and trial leadership (including study cochairs or subinvestigators) will also be expected to use conference speaking opportunities to discuss the science around the clinical trial and promote awareness about the study design and eligible population. For trials with industry funding available, additional investigator sessions could be held with an industry collaborator at appropriate scientific meetings such as the American Society of Clinical Oncology or the American Society for Radiation Oncology. As these investigator sessions are an additional cost, they are only included for trials with industry funding.

**Table 2:** Principal investigator tool kit—tools for the accrual phase.

Tasks	Responsible party	Product or placement
Study updates^a^	Protocol Development or PI^b^	NRG Oncology semiannual meeting
Trials-in-progress abstract	Study Chair or NRG Oncology publications	Conference poster at ASCO^c^ and disease-specific society meetings
Trial-related social media messages	Study Chair, Communications Committee, patient advocates, and other stakeholders	Monthly compilation of Twitter, Facebook, and other social media platform visibility
Mention trial in education sessions	Study Chair and NCTN^d^ study champions	Relevant professional meetings
Investigator luncheon	Industry partner	Relevant professional meetings
Monitor accrual	Study Chair	Review month CTSU^e^ reports

^a^Selected trials only.

^b^PI: principal investigator.

^c^ASCO: American Society of Clinical Oncology.

^d^NCTN: National Clinical Trials Network.

^e^CTSU: Clinical Trials Support Unit.

A vital component of the PI tool kit is the use of social media platforms such as Twitter. The study principal investigator with assistance from the Communications Committee members will be expected to engage with the scientific community through these platforms around the clinical trial and other scientific advances in the disease space that may be pertinent to the ongoing clinical trial. Public communication through social media about clinical trials is an important avenue to raise awareness of these trials with patients, caregivers, and patient advocacy groups, all of whom have a presence on social media platforms. Many patient-led disease-specific advocacy groups use social media platforms to engage with patient communities; through collaboration with an identified patient advocate, messaging about the importance and availability of clinical trials may be amplified within disease-specific communities [11,12]. Importantly, every trial should identify a patient advocate to collaborate with during the accrual period. This can be carried out through the NRG Oncology patient advocacy committee, which is a committee of patient advocates that works closely with NRG Oncology on all phases of clinical trial development.

During the trial accrual period, a critical monitoring tool to be used is monthly CTSU reports. These reports are sent to trial principal investigators. They outline the number of study sites that are approved to enroll study participants, as well as monthly and overall accrual reports (Multimedia Appendix 2).

### Tools to Address Barriers to Accrual

The PI tool kit includes specific tools for trials that are not meeting accrual goals (Table 3). The first tool includes a process for identifying barriers to patient accrual. This information can be gleaned from site principal investigators during monthly NRG Oncology disease-specific meetings. These touch points with site principal investigators are crucial for an understanding of major reasons for screen failure or patients declining trial participation. After such barriers are identified, subsequent adjustments or trial amendments can be made that address specific issues (if required). It is crucial that the study principal investigator maintains a high level of bidirectional communication with site principal investigators and the disease group such that any barriers to accrual can be identified and addressed in a timely manner.

For trials that are not meeting accrual targets, a patient landing page can be created that is located within the NRG Oncology website to provide study information to health care teams and patients in a seamless way. Other tools include site surveys to reengage the study teams and surveys that assess the feasibility of opening the study at other sites that are not currently open to accrual. NCTN study champions should also be engaged when accrual is lacking and to continue to energize the NCTN community around trial accrual. Moreover, monthly webinars with the study principal investigator can be used to facilitate engagement with site principal investigators to further enhance accrual efforts. Such engagement can be performed with monthly protocol webinars for active study sites. Additionally, each NCTN group has an oversight committee, which monitors study accrual and can help address accrual issues with the study chair of underperforming trials. Within NRG Oncology, the POM committee reviews accrual data quarterly for all NRG trials and connects with study principal investigators as needed to offer support for improving accrual.

**Table 3:** Principal investigator tool kit—trials experiencing accrual barriers.

Tasks	Responsible party	Product or placement
Identify barriers to accrual and adapt	Study Chair or Protocol Development team	Monthly site calls or amendments Webinar
Engage community intermediaries	Study Chair with support of disease-specific committee and advocates	Targeted communication about the trial
Regular contact with institutional PIs^a^	Study Chair with support from Protocol Development	Monthly calls or webinars In-person meetings
Consider study landing page (if not already available)	Protocol Development or Communications Committee with Study Chair review	NRG Oncology web page CTSU^b^ web page
Use NCTN^c^ champions (if not already available)	Study Chair with Protocol Development	Sponsorship of trial at other NCTN groups
Conduct site surveys	Study Chair	Assessment of feasibility of opening new sites or reengaging existing sites

^a^PIs: principal investigators.

^b^CTSU: Clinical Trials Support Unit.

^c^NCTN: National Clinical Trials Network.

### Piloting the Study Principal Investigator Tool Kit

The NRG Oncology PI tool kit was developed in 2019, with the idea that the tool kit would be piloted by a trial selected by the POM committee. NRG Oncology/Alliance NRG-LU005 launched on May 28, 2019. The latter is a trial for limited-stage, small-cell lung cancer that is testing standard chemoradiation with or without atezolizumab. This is a phase II or III study with a target accrual of 506 patients. Historically, clinical trials in limited-stage, small-cell lung cancer have been difficult to complete [13], and it was felt that the PI tool kit would be a valuable resource to use at the outset of NRG Oncology/Alliance NRG-LU005. The PI tool kit was used at study launch and throughout study accrual, which was completed on June 30, 2022.

## Results

**Figure 1:**
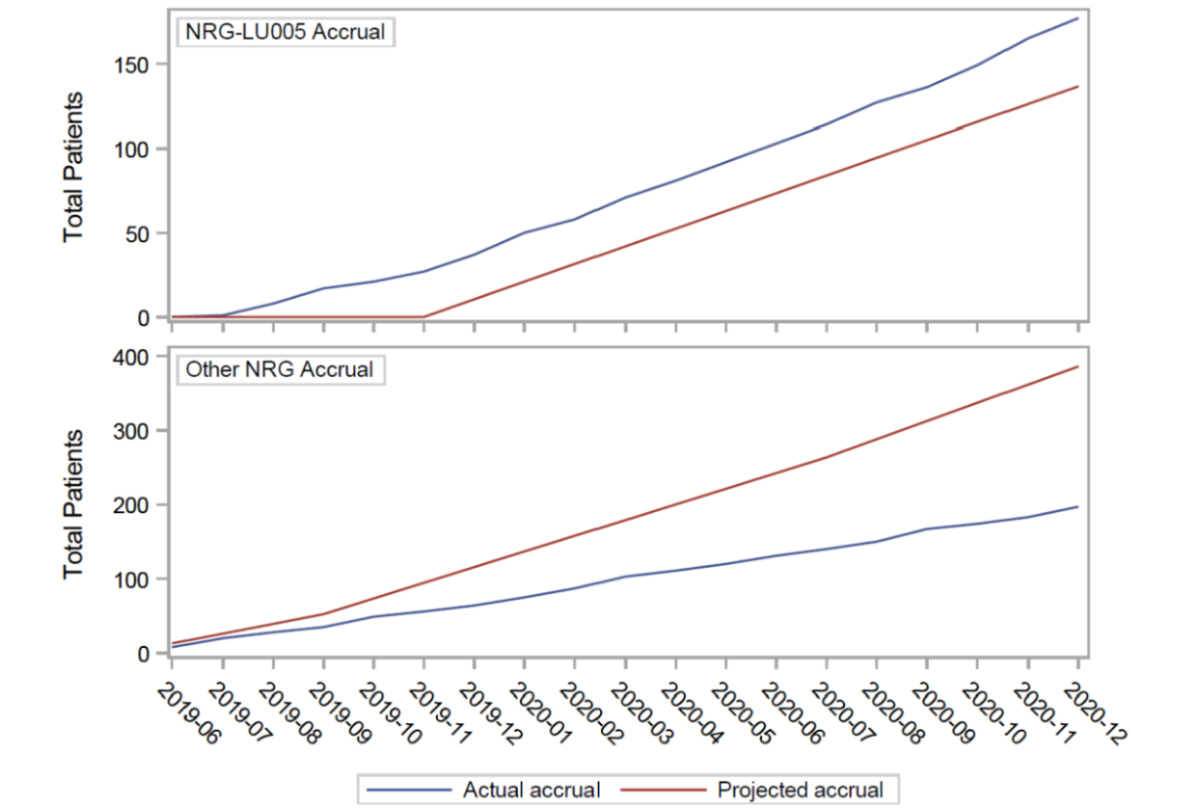
Accrual pace of NRG/Alliance LU005 (top panel) relative to other NRG Oncology lung cancer phase II or III trials (bottom panel).

NRG Oncology/Alliance NRG-LU005 has been accruing ahead of the projected schedule since the time of study launch. This has occurred despite the COVID-19, pandemic which began several months after the study launched (Figure 1). As of January 31, 2021, a total of 374 sites were approved for enrollment; 109 (29.1%) sites have accrued at least one patient, and 20 (5.3%) sites have accrued three patients or more. The trial was projected to accrue 10.5 patients per month; however, the accrual rate for the last 6 months has been 11.3 patients per month. As of October 2, 2021, total accrual was 127% of the projected accrual.

Compared with the rate of study accrual for Cancer and Leukemia Group B 30610, the prior NCTN study in limited-stage, small-cell lung cancer (activated on March 15, 2008), NRG Oncology/Alliance NRG-LU005 accrual, which used the PI tool kit intervention, was higher. The latter enrolled 48 patients during the first 9 months of activation, compared with 18 patients enrolled in Cancer and Leukemia Group B 30610 during the first 9 months of activation. These 2 trials enrolled the same patient population with very similar inclusion criteria, including newly diagnosed, limited-stage, small-cell lung cancer without prior treatment, with an Eastern Cooperative Group Performance Status of 0-2 [14]. Considering the accrual of NRG Oncology/Alliance NRG-LU005 relative to other NRG Oncology lung cancer phase II or III trials during the same period (June 2019 to December 2020), Figure 1 shows an accrual greater than what was projected for NRG Oncology/Alliance NRG-LU005, while other NRG Oncology lung cancer trials accrued at rates less than what was projected. Additionally, NRG Oncology/Alliance NRG-LU005 accrual was not negatively impacted by the COVID-19 pandemic relative to other NRG lung cancer trials, which did see reduced accrual with the onset of the pandemic in early 2020.

In terms of operationalizing the PI tool kit, NRG Oncology specifically assessed ongoing clinical trials for retroactive use of study PI tool kit elements. Out of the 71 active studies, 40 (56%) are currently using at least one element of the tool kit, with 29 studies (41%) using patient brochures, 24 (34%) with study training slides, 26 (37%) with social media cards, 9 (13%) with a study flyer, 6 (8%) with study newsletters, and 6 (8%) using study-specific webinars. Given the successful adoption of several PI tool kit elements in active studies and early feasibility of proactive tool kit use during NRG LU-005 activation, operationalizing the PI tool kit for all new NRG Oncology studies in development is not expected to be difficult or costly.

## Discussion

### Principal Findings

Accrual to federally funded NCTN trials is critical to advance cancer care and to study novel treatments. Many trials within the NCTN portfolio fail to accrue as rapidly as projected, and this can ultimately lead to delayed knowledge of treatment effect or study closure as well as poor use of limited resources. Premature closure of federally funded clinical trials ultimately results in tax-payer dollars being used in an ineffective way. A study of National Cancer Institute Cancer Therapy Evaluation Program phase I-III trials between 2000 and 2007 showed that 81.5% of trials did not achieve projected accrual goals, and 37.2% failed to achieve the minimum projected accrual at study closure [15]. This study also showed that trials that accrue the first patient beyond 2 months from activation are significantly less likely to achieve accrual goals [15]. Another study of NCI cooperative group phase III trials activated from 2000 to 2007 demonstrated that the number of phase-III trials that did not reach their projected total accrual due to insufficient enrollment was estimated to be 22% for pediatric and adult trials combined and 26.7% for nonpediatric trials [16]. Targeted interventions designed to optimize accrual early, at the time of activation and throughout the duration of the trial, are urgently needed to answer our most pressing scientific questions in oncology. Particularly considering the COVID-19 pandemic, with reduced enrollment in clinical trials across the United States, methods to help investigators overcome accrual barriers and a road map of resources offer the potential to address trial accrual barriers in a timely manner.

The NRG Oncology PI tool kit was developed to take a multipronged approach in a style of a checklist with accrual-enhancing activities developed and performed by both NRG Oncology staff and the study principal investigator. The PI tool kit uses a variety of methods to leverage optimal engagement within the scientific and patient advocate community. Engagement with the patient and physician population that will participate in the trial serves as the cornerstone of the PI tool kit, and it is expected that the study principal investigator or physician champions have an active and professional Twitter presence. By creating a clear road map of activities to be performed at key time points during the study lifetime, the study team can most optimally engage and support trial accrual. The PI tool kit was designed to be used for any cancer disease site and could be readily adopted by other groups within the NCTN. The PI tool kit was piloted with NRG Oncology/Alliance NRG-LU005, and this trial has exceeded accrual goals despite the COVID-19 pandemic. NRG Oncology plans to use the PI tool kit for all future phase II and III trials that are activated. Notably, most of the tools in the tool kit are low-cost and can be implemented with a modest degree of infrastructure.

### Comparison With Prior Work

Many of the interventions described in the tool kit use social media platforms for awareness and engagement. The role of social media in enhancing recruitment to clinical trials is an active area of investigation. Social media can be leveraged to engage with patients with cancer, including rare cancers, and facilitate patient knowledge of and enrollment to clinical trials [17]. A recent review reported that preliminary data suggest that social media platforms can enhance patient participation in a cost-effective manner [18]. However, there are currently barriers to generating high-quality, evidence-based data, specifically assessing how social media platforms impact clinical trial accrual, primarily due the difficulties in capturing data. It has also been suggested that social media platforms such as Facebook and Twitter may also be tools that could improve recruitment of minority populations to clinical trials [19]. In an evaluation of recruitment methods to a randomized controlled study of Spanish-speaking smokers in the United States, Facebook was the most effective method of recruitment for enrolling Hispanic or Latinx smokers [20].

### Limitations

One important caveat to the PI tool kit is that it primarily serves to amplify study accrual for well-designed clinical trials that are asking important scientific questions. The success in patient accrual seen in the first pilot trial (NRG Oncology/Alliance NRG-LU005) is also attributed to the excitement around immunotherapy in patients with small-cell lung cancer, given recent data showing a survival benefit when immunotherapy is combined with frontline chemotherapy in extensive-stage, small-cell lung cancer [21,22], which cannot be attributed to the PI tool kit alone.

While the NRG Oncology PI tool kit focuses primarily on tools that enhance engagement, there are certainly other tools that could be explored to improve accrual to clinical trials. The PI tool kit does not specifically address fundamental flaws in trial design that could negatively impact accrual. Additionally, it focuses on overall accrual and does not have any tools to enhance enrollment of patients from underrepresented groups and elderly patients, which is important for study applicability. Future efforts for NRG Oncology include the development of specific tools to enhance the accrual of diverse patient populations. Lastly, the PI tool kit is in the early phase of development, and as such, it has not been fully implemented into all NRG Oncology trials. Implementation science methodologies have not yet been used to fully integrate the tool kit into all clinical trials within our organization.

### Conclusions

In conclusion, the NRG Oncology PI tool kit was created to enhance overall accrual efforts to NCI-sponsored clinical trials. With a focus on tools that will enhance engagement across the stakeholders in oncology care, the PI tool kit fills an unmet need and could be widely adopted across the NCTN.

## Appendix

Multimedia Appendix 1 Principle Investigator tool kit instructions.

Multimedia Appendix 2 Site accrual summary.

## Abbreviations

CIRB: central institutional review board
CTSU: Clinical Trials Support Unit
NCI: National Cancer Institute
NCTN: National Clinical Trial Network
PI: principal investigator
POM: Protocol Operations Management

## Data Availability

Research data are stored in an institutional repository and will be shared upon request from the corresponding author.
