# Supplementary material for: Creating and Implementing a Principal Investigator Tool Kit for Enhancing Accrual to Late Phase Clinical Trials: Development and Usability Study
Source: JMIR Cancer. 2022 Aug 25;8(3):e38514. doi: 10.2196/38514 (PMC9459930; doi:10.2196/38514)
Supplement: Multimedia Appendix 1 [file cancer_v8i3e38514_app1.pdf]

NRG ONCOLOGY

Advancing Research. Improving Lives.™

Instructions for PI Toolkit (Study Promotion Checklist)

- A Promotion Checklist is created for each NRG study.
- The protocol administrator (PA) is responsible for sending out the Promotion Checklist to PIs at the time the protocol is given an NCI status of "Approval-on-Hold".
  - If a project manager (PM) is identified, the PA sends the unaltered checklist to the PI, copys the PM, and makes introductions. The PM is then responsible for customizing the checklist.
  - If there is no study PM, PAs work with the Director of PDRC to customize the promotion checklist before sending to PI.
  - Be sure to copy the NRG Protocol Operations Management (POM) committee liaison, Disease Site Chair, POM Chair and POM Administrator (Sally Bialy) when sending to PI.
- The PA/PM must hold a call with the study chair(s) along with the POM liaison to discuss the promotion checklist and expectations.
- ***Each study must at a minimum have a study overview slide set and patient brochure.***
- All checklist items are at the investigator's discretion, and the investigator will work with the POM Committee liaison to utilize the PI toolkit.
- The PA/PM may use the checklist to add notes and dates of completion for items on the checklist.
- File customized Promotion Checklists here for NRG Ops West studies \\acr.org\shares\RTOG\RTOG Teams\Study Promotion PI Checklists. A blank form is also there.
  - For Breast, GYN and Colorectal a copy of the checklist should also be sent to NRG Ops West for central filing. This central filing is for POM committee use. Please send to Nancy Soto.

<u>During Study Conduct</u>:

- If Statistics or POM alerts the study team that the study is underperforming, the PA/PM should review the checklist with the PI to discuss implementation of other appropriate checklist items.

NRG ONCOLOGY

Advancing Research. Improving Lives.™

The following table outlines the study promotions that can be done for your trial. As Study PI, you are responsible for completing some of these tasks on your own while others are supported by NRG staff. Please review the list carefully and congratulations on your study activation and may it accrue quickly and easily.

Study Number/Title/PI:

Date:

**Item**	**Before or At Study Activation**	**Comments**
**Study Overview Slide Set**<br>- ***PD or Project Manager drafts***<br>- Posted on CTSU website (NRG, if applicable)	Required *before* activation	Study team and PI reviews / approves<br>Note: Update for amendments, as needed
**Patient Brochure**<br>- PD alerts Communications to create<br>- PD submits to CIRB<br>- Posted to CTSU and NRG websites	Required *before* activation	Study PI reviews / approves<br>Patient Advocate and Health Disparties Chairs can be consultated to ensure and to encourage Diversity/Inclusion content<br>Needs CIRB approval prior to posting<br>Note: Update for amendments, as needed
**Letter to site investigators**<br>- Study PI drafts email and sends<br>- Work with POM liaison on text	Required *at* activation; may be repeated during study, if needed	Site PI Emails can be obtained either from NRG Members services or CTSU promotions team speak w/***PD or Project Manager***<br>Note: Study Statisticians can provide a site list based on previous studies, if applicable
**Social Media (video, tweets)**<br>- Study PI Work w/POM liaison and Communications	Required	Who can tweet: Study PI/sub-investigators, NRG Communications, Patient Advocates<br>**Attend the NRG Social Media Workshop (held at NRG Meetings)**<br>Example tweets: study activation, first patient in.
**Kick off session/webinar** ****select trials only****<br>- ***PD/Project Manager or NCORP Administrator will help coordinate***	Required *at* activation	***PD/Project Manager or NCORP Administrator*** handles logistics<br>Study PI and Team review /approve and present slides<br>Plan around NRG Semiannual Meetings, *preferred*
**Study Landing Page for patients** ****select trials only****<br>- Work w/Communications, POM liaison and Patient Advocate	Optional *at* activation or during study	Communications team handles logistics<br>Study PI reviews / approves content aligns with the protocol<br>Patient Advocate reviews content

NRG ONCOLOGY

Advancing Research. Improving Lives.™

Item	During the Study	Comments
**Monitor Accrual (Monthly)**<br>- CTSU sends monthly report (except for phase I studies)<br>- Study statisticians also provide accrual reports	Required	Study PI reviews both CTSU and NRG accrual reports<br>Highly recommended Review of CTSU study-specific webpage (often)<br>Work w/***PD team or Project Manager on any action items needed.***
**Study Update sessions*select trials only***<br>- ***PD/Project Manager or NCORP Administrator will help coordinate***	Required	***PD team or Project Manager or NCORP Administrator*** handles logistics<br>Study PI and Team review /approve and present slides<br>Plan around NRG Semiannual Meetings, *preferred*
**Trials in progress abstract**<br>(i.e., ASCO, ASTRO)	Required	• Study PI must create in conjunction with study statistician (organizations have different format requirements for submission, please check). Study statistician or study PI submits draft to NRG Publications.<br>• NRG Publications reviews & approves prior to submission, including obtaining CTEP and/or collaborator review/approval.<br>• Study chair submits by conference deadline.<br>• Presents at time of conference.<br>• Refer to NRG Oncology Publications Policy and Guidelines https://www.nrgoncology.org/Clinical-Trials/Publications
**Social Media**	Required	Who can tweet: Study PI/sub-investigators, NRG Communications, Patient Advocates<br>**Attend the NRG Social Media Workshop (held at NRG Meetings)**<br>Example tweets: enrollment milestones, Trials in progress Abstract, upcoming study webinar, etc.
**Education Sessions**	*At any opportunity*	Study PI/sub-investigators can present (use study overview slides)<br>**NCTN Champion presents at Lead Protocol Organization group meetings**<br>At time of a conference
**NRG study newsletters *select trials only***	*Optional, as needed*	Study PI drafts content. ***PD or Project Manager or NCORP Administrator*** assists with team review and sends to Communications<br>Communications does lay out and placement onto NRG newsletter

Note: the Item cell of the last row also contains: - Work w/***Project Manager or NCORP Administrator*** and Communications

04Mar2022

**Investigator Meeting*select trials only***	*At any opportunity*	If industry collaborator supports<br>Study PI/sub-investigators can present (use study overview slides)<br>At time of conference

For trials experiencing accrual barriers after the above has been implemented. The following will be recommended by the Protocol Operations & Management (POM) committee liaison:

- **Monthly site calls to identify accrual barriers and/or to engage community**
- **Conduct site surveys**
- **Amend study if required**
- **Considers Study landing page and/or other study materials.**
- **Opportunities for consultation (e.g NCI DIEG Research Studios Referral)**

Resources

FORCE TIP Sheet for Researchers: https://www.facingourrisk.org/uploads/Tip-Sheet-For-Researchers-Final-04.10.2021.pdf

NRG Contact Information	
**Protocol Development Contact**:	Name and contact information (phone and email)
**Project Manager, if applicable**	Name and contact information (phone and email)
**Communications Contact:**	Name and contact information (phone and email)
**Protocol Operations & Management (POM) Committee Liaison** ***(Add liaison prior to sending and copy on email. Liaisons are assigned by POM Chair/Co-Chair; ask Director of PD for liaison)***	Name and contact information (phone and email)
Include anyone else, as needed or assigned to help with enrollment (Example, Patient Advocate liaison, NCORP Administrator/liaison, Health Disparities liaison, CCD, etc.)	
