# Supplementary material for: Creating and Implementing a Principal Investigator Tool Kit for Enhancing Accrual to Late Phase Clinical Trials: Development and Usability Study
Source: JMIR Cancer. 2022 Aug 25;8(3):e38514. doi: 10.2196/38514 (PMC9459930; doi:10.2196/38514)
Supplement: Multimedia Appendix 2 [file cancer_v8i3e38514_app2.pdf]

Site Accrual Summary

#	Site	Site Name	Site Initial Approval Date	Accrual Total	Date of First Accrual	Date of Last Accrual
1	WV025	West Virginia University Healthcare	15-Jul-2020	3	26-Jul-2021	24-Mar-2022
2	WI212	ThedaCare Regional Cancer Center	09-Nov-2020	5	01-Jun-2021	16-Jun-2022
3	WI210	Marshfield Medical Center-River Region at Stevens Point	21-Jul-2021	1	21-Jul-2021	21-Jul-2021
4	WI196	Westfields Hospital/Cancer Center of Western Wisconsin	13-Aug-2020	1	20-Aug-2020	20-Aug-2020
5	WI156	Vince Lombardi Cancer Clinic - Oshkosh	09-Mar-2020	1	27-Jan-2021	27-Jan-2021
6	WI144	Marshfield Medical Center - Weston	26-Feb-2020	3	02-Dec-2020	09-Aug-2021
7	WI114	Aurora Cancer Care -Southern Lakes VLCC	09-Mar-2020	1	17-Jul-2020	17-Jul-2020
8	WI087	ProHealth Oconomowoc Memorial Hospital	09-Jun-2021	1	26-Nov-2021	26-Nov-2021

Site Accrual Summary

#	Site	Site Name	Site Initial Approval Date	Accrual Total	Date of First Accrual	Date of Last Accrual
9	WI029	Gundersen Lutheran Medical Center	16-Jul-2019	2	01-Apr-2020	04-Nov-2021
10	WI028	Aspirus Regional Cancer Center	22-Jul-2019	5	05-Oct-2020	03-Sep-2021
11	WI020	University of Wisconsin Carbone Cancer Center	30-Sep-2019	6	27-Jan-2020	08-Jun-2022
12	WI011	Aurora Saint Luke's Medical Center	09-Mar-2020	6	12-Aug-2020	23-Jun-2022
13	WI004	Zablocki Veterans Administration Medical Center	04-Nov-2019	1	04-Mar-2022	04-Mar-2022
14	WA076	Providence Regional Cancer System-Aberdeen	05-Sep-2019	1	16-Sep-2019	16-Sep-2019
15	VT003	University of Vermont Medical Center	22-Jan-2020	4	21-Feb-2020	09-Dec-2021
16	VA188	Augusta Health Center for Cancer and Blood Disorders	31-Jul-2019	3	25-Sep-2019	11-Nov-2020

Site Accrual Summary

#	Site	Site Name	Site Initial Approval Date	Accrual Total	Date of First Accrual	Date of Last Accrual
17	VA010	Virginia Commonwealth University/ Massey Cancer Center	19-Oct-2020	1	10-Mar-2022	10-Mar-2022
18	VA009	University of Virginia Cancer Center	05-Sep-2019	4	05-Sep-2019	01-Sep-2021
19	UT003	Huntsman Cancer Institute/University of Utah	27-Jan-2020	5	23-Jun-2020	04-Feb-2022
20	TX458	Houston Methodist West Hospital	07-Jul-2020	1	08-Jul-2020	08-Jul-2020
21	TX455	Houston Methodist The Woodlands Hospital	18-May-2021	4	18-May-2021	24-Jan-2022
22	TX454	Houston Methodist Saint John Hospital	22-Jun-2022	1	23-Jun-2022	23-Jun-2022
23	TX411	Houston Methodist Sugar Land Hospital	13-Oct-2020	2	05-May-2021	22-Jun-2021
24	TX403	MD Anderson League City	14-Apr-2021	1	12-Oct-2021	12-Oct-2021
25	TX401	MD Anderson in Sugar Land	14-Apr-2021	2	11-Aug-2021	27-Sep-2021
26	TX400	MD Anderson West Houston	14-Apr-2021	3	25-Nov-2021	23-Jun-2022

Site Accrual Summary

#	Site	Site Name	Site Initial Approval Date	Accrual Total	Date of First Accrual	Date of Last Accrual
27	TX036	Houston Methodist Hospital	01-Apr-2021	1	07-Dec-2021	07-Dec-2021
28	TX035	M D Anderson Cancer Center	10-Jul-2020	9	22-Aug-2020	07-Jun-2022
29	TN038	Thompson Cancer Survival Center	27-Jun-2019	2	10-Dec-2020	19-Apr-2022
30	TN029	Baptist Memorial Hospital and Cancer Center-Memphis	04-Dec-2020	1	24-Mar-2022	24-Mar-2022
31	SD006	Monument Health Rapid City Hospital	21-Nov-2019	2	31-Jul-2020	20-Aug-2020
32	SC117	Saint Joseph's/Candler - Bluffton Campus	27-May-2021	1	30-Sep-2021	30-Sep-2021
33	SC036	Prisma Health Cancer Institute - Eastside	18-Jun-2020	1	19-May-2022	19-May-2022
34	SC024	Spartanburg Medical Center	18-Nov-2019	3	20-Dec-2019	09-Sep-2021
35	SC008	Medical University of South Carolina	30-Apr-2021	1	24-May-2022	24-May-2022
36	RI005	Rhode Island Hospital	31-Dec-2019	7	10-Jan-2020	10-Nov-2021

Site Accrual Summary

#	Site	Site Name	Site Initial Approval Date	Accrual Total	Date of First Accrual	Date of Last Accrual
37	PA491	Crozer-Keystone Regional Cancer Center at Broomall	25-Jan-2021	1	25-Jan-2021	25-Jan-2021
38	PA476	Lancaster General Ann B Barshinger Cancer Institute	27-Oct-2020	2	07-Apr-2021	20-May-2021
39	PA429	Adams Cancer Center	04-Sep-2019	1	13-Oct-2021	13-Oct-2021
40	PA406	UPMC Cancer Center at UPMC Horizon	17-Mar-2022	3	21-Mar-2022	27-May-2022
41	PA254	WellSpan Health-York Cancer Center	04-Sep-2019	4	11-Sep-2019	10-Dec-2021
42	PA125	Lankenau Medical Center	11-Oct-2019	3	18-Dec-2019	15-Dec-2020
43	PA124	Paoli Memorial Hospital	11-Oct-2019	2	22-Nov-2021	04-Apr-2022
44	PA121	Thomas Jefferson University Hospital	18-Oct-2019	3	30-Jan-2020	26-Oct-2021
45	PA062	Guthrie Medical Group PC-Robert Packer Hospital	29-Jul-2019	3	13-Sep-2019	20-Jun-2022
46	PA055	Lehigh Valley Hospital-Cedar Crest	16-Mar-2020	1	29-May-2020	29-May-2020
47	PA052	Geisinger Medical Center	18-Nov-2020	3	18-Nov-2020	08-Jun-2022

Site Accrual Summary

#	Site	Site Name	Site Initial Approval Date	Accrual Total	Date of First Accrual	Date of Last Accrual
48	PA015	UPMC Hillman Cancer Center	29-Dec-2021	1	07-Feb-2022	07-Feb-2022
49	OR013	Legacy Good Samaritan Hospital and Medical Center	08-Jul-2019	3	13-May-2020	09-Nov-2021
50	OK010	Mercy Hospital Oklahoma City	06-Jul-2020	1	13-Jul-2020	13-Jul-2020
51	OK003	University of Oklahoma Health Sciences Center	24-Jul-2019	1	18-Dec-2019	18-Dec-2019
52	OH394	University of Cincinnati Cancer Center-West Chester	04-Nov-2019	3	12-Nov-2020	11-Oct-2021
53	OH314	North Coast Cancer Care	19-Feb-2020	1	11-Jan-2022	11-Jan-2022
54	OH248	UHHS-Westlake Medical Center	30-Oct-2019	1	21-Feb-2020	21-Feb-2020
55	OH184	Geauga Hospital	12-Dec-2019	2	09-Apr-2020	29-Oct-2021
56	OH183	UH Seidman Cancer Center at Lake Health Mentor Campus	24-Oct-2019	1	21-Mar-2022	21-Mar-2022
57	OH182	Adena Regional Medical Center	16-Jul-2019	3	26-Aug-2020	27-Jan-2021

Site Accrual Summary

#	Site	Site Name	Site Initial Approval Date	Accrual Total	Date of First Accrual	Date of Last Accrual
58	OH100	Aultman Health Foundation	31-Oct-2019	1	09-Apr-2021	09-Apr-2021
59	OH097	Hillcrest Hospital Cancer Center	26-Feb-2020	1	17-Mar-2022	17-Mar-2022
60	OH070	University of Cincinnati Cancer Center-UC Medical Center	04-Nov-2019	2	21-Aug-2020	13-Oct-2020
61	OH055	Summa Health System - Akron Campus	01-Oct-2019	1	07-May-2021	07-May-2021
62	OH045	UH Seidman Cancer Center at Southwest General Hospital	24-Oct-2019	1	10-May-2022	10-May-2022
63	OH038	Cleveland Clinic Cancer Center/Fair view Hospital	19-Feb-2020	4	27-Apr-2020	01-Jul-2021
64	OH036	MetroHealt h Medical Center	05-May-2020	2	05-Nov-2020	19-Jul-2021
65	OH029	Case Western Reserve University	24-Oct-2019	7	23-Oct-2020	30-Mar-2022
66	OH027	Cleveland Clinic Foundation	18-Feb-2020	2	14-Aug-2020	11-Mar-2021

Site Accrual Summary

#	Site	Site Name	Site Initial Approval Date	Accrual Total	Date of First Accrual	Date of Last Accrual
67	OH007	Ohio State University Comprehensive Cancer Center	21-Aug-2019	18	17-Sep-2019	24-May-2022
68	NY367	Arnot Ogden Medical Center/Falck Cancer Center	12-Jul-2019	1	16-Aug-2019	16-Aug-2019
69	NY358	Mount Sinai Chelsea	25-Mar-2021	2	16-Apr-2021	24-May-2021
70	NY313	Montefiore Medical Center-Einstein Campus	04-Nov-2019	4	24-Jan-2020	20-May-2022
71	NY167	University of Rochester	08-Jan-2020	10	23-Dec-2020	24-May-2022
72	NY134	Glens Falls Hospital	13-Jan-2020	2	22-Jan-2020	27-Apr-2020
73	NY045	Montefiore Medical Center - Moses Campus	04-Nov-2019	1	13-Oct-2021	13-Oct-2021
74	NY021	Mount Sinai Hospital	25-Mar-2021	1	10-May-2021	10-May-2021
75	NM004	University of New Mexico Cancer Center	13-Nov-2019	4	25-Jun-2020	28-Feb-2022
76	NJ099	Southern Ocean County Medical Center	29-Mar-2021	1	12-Apr-2021	12-Apr-2021

Site Accrual Summary

#	Site	Site Name	Site Initial Approval Date	Accrual Total	Date of First Accrual	Date of Last Accrual
77	NJ066	Rutgers Cancer Institute of New Jersey	18-Jun-2020	3	29-Jun-2020	24-Mar-2022
78	NH038	New Hampshire Oncology Hematology PA-Concord	20-Sep-2019	1	25-May-2022	25-May-2022
79	NH015	Solinsky Center for Cancer Care	20-Sep-2019	1	07-Aug-2020	07-Aug-2020
80	NH012	Dartmouth-Hitchcock Medical Center/Norris Cotton Cancer Center	10-Dec-2020	5	05-Mar-2021	23-Jun-2022
81	NE073	Cancer Partners of Nebraska - Pine Lake	06-May-2021	1	14-Apr-2022	14-Apr-2022
82	NE036	Cancer Partners of Nebraska	27-Jan-2021	2	06-May-2021	22-Apr-2022
83	NE025	Alegent Health Immanuel Medical Center	25-Mar-2020	1	25-Mar-2020	25-Mar-2020
84	NE017	Alegent Health Bergan Mercy Medical Center	25-Oct-2019	1	11-Nov-2019	11-Nov-2019
85	NE007	Nebraska Methodist Hospital	13-Sep-2019	1	20-Oct-2021	20-Oct-2021

Site Accrual Summary

#	Site	Site Name	Site Initial Approval Date	Accrual Total	Date of First Accrual	Date of Last Accrual
86	NE003	University of Nebraska Medical Center	05-Dec-2021	1	15-Dec-2021	15-Dec-2021
87	ND028	Altru Cancer Center	12-Aug-2019	1	29-Aug-2019	29-Aug-2019
88	ND005	Sanford Roger Maris Cancer Center	16-Dec-2019	2	16-Dec-2020	27-Oct-2021
89	NC090	Margaret R Pardee Memorial Hospital	06-Jul-2019	2	17-Apr-2020	12-Apr-2021
90	NC083	Atrium Health Cabarrus/LCI-Concord	06-Apr-2021	1	01-Jun-2022	01-Jun-2022
91	NC081	FirstHealth of the Carolinas-Moore Regional Hospital	10-Jul-2019	9	06-Dec-2019	09-Jun-2022
92	NC053	Messino Cancer Centers	16-Mar-2021	2	23-Sep-2021	07-Oct-2021
93	NC042	Carolinas Medical Center/Levine Cancer Institute	06-Apr-2021	2	01-Mar-2022	25-Mar-2022
94	NC019	Novant Health Presbyterian Medical Center	11-Nov-2021	1	23-Jun-2022	23-Jun-2022
95	NC004	Cone Health Cancer Center	22-Jul-2019	1	25-Feb-2022	25-Feb-2022

Site Accrual Summary

#	Site	Site Name	Site Initial Approval Date	Accrual Total	Date of First Accrual	Date of Last Accrual
96	NC002	Wake Forest University Health Sciences	20-Dec-2019	7	05-Jun-2020	03-Aug-2021
97	MT019	Bozeman Health Deaconess Hospital	22-Jan-2021	1	17-Jun-2022	17-Jun-2022
98	MS056	Baptist Memorial Hospital and Cancer Center-Oxford	04-Dec-2020	1	03-Jun-2021	03-Jun-2021
99	MS055	Baptist Memorial Hospital and Cancer Center-Desoto	04-Dec-2020	2	16-Apr-2021	18-Oct-2021
100	MO199	Parkland Health Center - Farmington	14-Sep-2020	2	21-Sep-2020	13-Apr-2021
101	MO152	Siteman Cancer Center at Saint Peters Hospital	08-Jan-2020	1	07-Jul-2021	07-Jul-2021
102	MO132	University of Kansas Cancer Center - Lee's Summit	17-Sep-2019	1	07-Oct-2020	07-Oct-2020
103	MO053	Siteman Cancer Center at West County Hospital	08-Jan-2020	1	25-Mar-2021	25-Mar-2021

Site Accrual Summary

#	Site	Site Name	Site Initial Approval Date	Accrual Total	Date of First Accrual	Date of Last Accrual
104	MO046	Missouri Baptist Medical Center	30-Jul-2019	5	09-Aug-2019	28-Jan-2022
105	MO011	Washington University School of Medicine	08-Jan-2020	4	23-Mar-2020	13-Apr-2021
106	MO009	Saint Francis Medical Center	25-Jul-2019	2	13-Jul-2020	12-Oct-2021
107	MN145	Minnesota Oncology - Burnsville	12-Apr-2022	1	12-Apr-2022	12-Apr-2022
108	MN079	Mayo Clinic Health Systems-Mankato	16-Mar-2020	1	01-Apr-2021	01-Apr-2021
109	MN075	Minnesota Oncology Hematology PA-Maplewood	03-Feb-2020	3	25-Feb-2020	22-Apr-2021
110	MN026	Mayo Clinic	06-Nov-2019	2	13-Apr-2021	15-Apr-2021
111	MN014	Park Nicollet Clinic - Saint Louis Park	19-Dec-2019	2	18-Mar-2021	11-Nov-2021
112	MN001	Regions Hospital	05-Feb-2020	1	24-May-2022	24-May-2022
113	MI372	GenesisCare USA - Macomb	23-Jul-2020	2	24-Nov-2020	08-Apr-2021
114	MI364	Ascension Saint Joseph Hospital	26-Aug-2021	1	21-Sep-2021	21-Sep-2021
115	MI327	Chelsea Hospital	13-Aug-2019	2	19-Mar-2020	17-Sep-2021

Site Accrual Summary

#	Site	Site Name	Site Initial Approval Date	Accrual Total	Date of First Accrual	Date of Last Accrual
116	MI323	GenesisCare USA - Troy	14-Jun-2019	2	25-Mar-2020	31-Jul-2020
117	MI310	GenesisCare USA - Farmington Hills	24-Jun-2019	1	06-Aug-2019	06-Aug-2019
118	MI294	Trinity Health Medical Center - Canton	13-Aug-2019	2	14-Dec-2020	26-Mar-2021
119	MI285	McLaren Cancer Institute-Clarkston	07-Oct-2019	1	25-May-2021	25-May-2021
120	MI269	Mercy Health Mercy Campus	03-Dec-2021	3	09-Dec-2021	28-Jan-2022
121	MI262	Singh and Arora Hematology Oncology PC	07-Oct-2019	2	08-Jan-2020	19-Mar-2021
122	MI221	Trinity Health Medical Center - Brighton	13-Aug-2019	3	15-Sep-2020	17-Jun-2022
123	MI132	Spectrum Health at Butterworth Campus	18-Aug-2021	2	06-Apr-2022	19-Apr-2022
124	MI131	Henry Ford Cancer Institute-Downriver	01-Mar-2022	1	15-Mar-2022	15-Mar-2022
125	MI128	William Beaumont Hospital - Troy	23-Jan-2020	1	17-Mar-2021	17-Mar-2021

Site Accrual Summary

#	Site	Site Name	Site Initial Approval Date	Accrual Total	Date of First Accrual	Date of Last Accrual
126	MI108	Lakeland Medical Center Saint Joseph	01-Nov-2019	3	01-Nov-2019	10-Nov-2021
127	MI104	GenesisCare USA - Madison Heights	22-Jul-2020	2	26-Feb-2021	03-Jun-2022
128	MI082	McLaren-Port Huron	07-Oct-2019	1	13-Apr-2022	13-Apr-2022
129	MI080	West Michigan Cancer Center	11-Oct-2019	1	20-Jan-2020	20-Jan-2020
130	MI079	Allegiance Health	05-Mar-2020	2	22-Oct-2020	23-Nov-2020
131	MI039	Sparrow Hospital	30-Sep-2019	3	16-Oct-2019	18-May-2022
132	MI037	McLaren Cancer Institute-Bay City	07-Oct-2019	1	15-Jul-2020	15-Jul-2020
133	MI026	Henry Ford Hospital	19-Sep-2019	2	27-Oct-2020	02-Mar-2021
134	MI017	Trinity Health Saint Mary Mercy Livonia Hospital	15-Oct-2021	1	23-May-2022	23-May-2022
135	MI013	Trinity Health Saint Joseph Mercy Hospital Ann Arbor	13-Aug-2019	8	03-Sep-2019	23-Jun-2022
136	MI005	William Beaumont Hospital-Royal Oak	23-Jan-2020	1	12-Jul-2021	12-Jul-2021

Site Accrual Summary

#	Site	Site Name	Site Initial Approval Date	Accrual Total	Date of First Accrual	Date of Last Accrual
137	ME035	MaineHealth/SMHC Cancer Care and Blood Disorders-Sanford	05-Aug-2019	1	28-May-2021	28-May-2021
138	MD157	UM Upper Chesapeake Medical Center	22-Jul-2020	3	03-Aug-2020	02-Nov-2021
139	MD027	Saint Agnes Hospital	27-Nov-2019	2	05-Dec-2019	03-Mar-2020
140	MD015	University of Maryland/Greenebaum Cancer Center	23-Aug-2021	1	15-Jun-2022	15-Jun-2022
141	MA036	Dana-Farber/Harvard Cancer Center	13-Dec-2019	7	04-Mar-2020	26-May-2022
142	MA011	UMass Memorial Medical Center - University Campus	21-Oct-2019	1	12-May-2021	12-May-2021
143	LA074	Our Lady of the Lake Physician Group	26-Nov-2019	2	05-Mar-2020	29-Apr-2021
144	LA007	Ochsner Medical Center Jefferson	12-Jun-2019	2	31-Dec-2019	17-Jun-2021
145	KY105	Norton Audubon Hospital and Medical Campus	01-Nov-2019	2	20-Jan-2021	22-Mar-2022

Site Accrual Summary

#	Site	Site Name	Site Initial Approval Date	Accrual Total	Date of First Accrual	Date of Last Accrual
146	KY100	Norton Brownsboro Hospital and Medical Campus	01-Nov-2019	2	02-Jan-2020	06-Feb-2020
147	KY049	Norton Hospital Pavilion and Medical Campus	01-Nov-2019	1	16-Jun-2022	16-Jun-2022
148	KY024	Baptist Health Louisville	14-Sep-2020	1	20-Jun-2022	20-Jun-2022
149	KY015	Saint Elizabeth Medical Center South	21-Oct-2020	2	13-May-2021	28-May-2021
150	KY002	The James Graham Brown Cancer Center at University of Louisville	16-Mar-2020	3	26-May-2021	11-Nov-2021
151	KS088	University of Kansas Hospital-Westwood Cancer Center	11-Sep-2019	1	19-May-2020	19-May-2020
152	KS055	Ascension Via Christi Hospitals Wichita	22-Oct-2019	5	31-Oct-2019	05-Apr-2021
153	KS029	Salina Regional Health Center	03-Feb-2021	2	16-Apr-2021	15-Jun-2021
154	IN181	Parkview Regional Medical Center	07-Aug-2019	2	09-Jan-2020	25-Jan-2022

Site Accrual Summary

#	Site	Site Name	Site Initial Approval Date	Accrual Total	Date of First Accrual	Date of Last Accrual
155	IN162	Franciscan Health Mooresville	18-Jun-2020	2	15-Jul-2021	19-Jul-2021
156	IN156	Community Cancer Center East	02-Dec-2020	4	05-Apr-2021	29-Nov-2021
157	IN087	Franciscan Health Indianapolis	18-Jun-2020	3	14-Sep-2021	18-May-2022
158	IN007	Indiana University/ Melvin and Bren Simon Cancer Center	20-May-2020	2	22-Mar-2021	09-Jun-2022
159	IN006	Community Cancer Center North	02-Dec-2020	1	23-Nov-2021	23-Nov-2021
160	IL208	Crossroads Cancer Center	19-Jun-2019	1	19-Nov-2020	19-Nov-2020
161	IL206	Elmhurst Memorial Hospital	07-Oct-2019	7	21-Feb-2020	13-Dec-2021
162	IL168	Carle Cancer Center	27-Jun-2019	2	13-Sep-2021	16-Mar-2022
163	IL120	Midwestern Regional Medical Center	09-Sep-2019	4	13-Mar-2020	22-Jun-2022
164	IL105	Ingalls Memorial Hospital	08-Mar-2021	2	26-Mar-2021	28-May-2021
165	IL104	Edward Hospital/Cancer Center	07-Oct-2019	2	22-Jul-2021	24-Sep-2021
166	IL101	Illinois CancerCare -Peoria	23-Jun-2019	1	07-Feb-2020	07-Feb-2020

Site Accrual Summary

#	Site	Site Name	Site Initial Approval Date	Accrual Total	Date of First Accrual	Date of Last Accrual
167	IL097	Springfield Memorial Hospital	01-Jul-2019	13	08-Nov-2019	27-May-2022
168	IL094	Decatur Memorial Hospital	19-Jun-2019	4	26-Jul-2019	28-Jul-2021
169	IL082	OSF Saint Francis Medical Center	23-Jun-2019	3	05-Aug-2019	25-Oct-2021
170	IL043	Rush University Medical Center	10-Jan-2020	2	18-Aug-2021	19-Nov-2021
171	IL040	University of Illinois	07-Aug-2019	2	30-Aug-2019	22-Jul-2020
172	IL036	Northwestern University	05-Feb-2020	2	13-Feb-2020	04-Sep-2020
173	IL017	Loyola University Medical Center	06-Jun-2020	1	14-Jan-2021	14-Jan-2021
174	IL004	NorthShore University HealthSystem-Highland Park Hospital	18-Apr-2022	1	26-Apr-2022	26-Apr-2022
175	ID011	Saint Alphonsus Cancer Care Center-Boise	21-Nov-2019	1	25-Nov-2019	25-Nov-2019
176	ID009	Saint Alphonsus Cancer Care Center-Nampa	21-Nov-2019	2	18-Mar-2020	09-Apr-2021

Site Accrual Summary

#	Site	Site Name	Site Initial Approval Date	Accrual Total	Date of First Accrual	Date of Last Accrual
177	IA066	Physicians' Clinic of Iowa PC	30-Nov-2020	1	19-Oct-2021	19-Oct-2021
178	IA024	Oncology Associates at Mercy Medical Center	30-Apr-2020	2	19-Jan-2022	25-Jan-2022
179	IA020	Mercy Hospital	30-Apr-2020	2	18-Nov-2020	29-Dec-2020
180	IA003	McFarland Clinic PC - Ames	18-Jun-2019	3	24-Oct-2019	28-Feb-2022
181	HI005	Queen's Medical Center	04-Nov-2020	1	22-Mar-2022	22-Mar-2022
182	GA229	Piedmont Fayette Hospital	11-Sep-2020	2	22-Oct-2020	12-May-2021
183	GA106	Lewis Cancer and Research Pavilion at Saint Joseph's/Candler	22-Oct-2019	1	19-Mar-2021	19-Mar-2021
184	GA005	Emory University Hospital/Winship Cancer Institute	07-Aug-2019	5	24-Apr-2020	01-Apr-2022
185	GA003	Grady Health System	07-Aug-2019	3	22-Jan-2021	04-Dec-2021
186	FL114	The Watson Clinic	17-Sep-2019	1	24-Jan-2022	24-Jan-2022
187	FL105	Sacred Heart Hospital	04-May-2021	1	05-Jan-2022	05-Jan-2022
188	FL078	Miami Cancer Institute	25-Mar-2020	11	27-Mar-2020	16-Jun-2022

Site Accrual Summary

#	Site	Site Name	Site Initial Approval Date	Accrual Total	Date of First Accrual	Date of Last Accrual
189	FL065	Moffitt Cancer Center	10-Jan-2020	7	20-Mar-2020	25-Feb-2022
190	FL028	University of Miami Miller School of Medicine-Sylvester Cancer Center	06-Jan-2021	1	15-Oct-2021	15-Oct-2021
191	FL020	Orlando Health Cancer Institute	28-Aug-2019	2	08-Oct-2021	11-Mar-2022
192	FL015	University of Florida Health Science Center - Gainesville	08-Aug-2019	2	28-Aug-2019	05-Sep-2019
193	DE028	Helen F Graham Cancer Center	25-Sep-2019	4	12-Jun-2020	10-Jun-2022
194	CO122	Parker Adventist Hospital	08-Dec-2021	1	22-Dec-2021	22-Dec-2021
195	CO070	University of Colorado Hospital	08-Mar-2020	1	29-Nov-2021	29-Nov-2021
196	CO029	UCHealth Memorial Hospital Central	04-Mar-2020	1	26-May-2020	26-May-2020
197	CO021	Penrose-Saint Francis Healthcare	15-Aug-2019	1	02-Oct-2020	02-Oct-2020
198	CO019	Poudre Valley Hospital	04-Mar-2020	1	25-Jun-2021	25-Jun-2021

Site Accrual Summary

#	Site	Site Name	Site Initial Approval Date	Accrual Total	Date of First Accrual	Date of Last Accrual
199	CA189	University of California Davis Comprehensive Cancer Center	18-Nov-2019	3	25-Nov-2019	02-Jun-2022
200	CA043	City of Hope Comprehensive Cancer Center	10-Jun-2021	1	01-Dec-2021	01-Dec-2021
201	CA011	USC / Norris Comprehensive Cancer Center	03-Aug-2020	2	14-Dec-2020	13-Apr-2021
202	AZ134	Banner MD Anderson Cancer Center	17-May-2021	2	18-Aug-2021	01-Feb-2022
203	AZ017	Banner University Medical Center - Tucson	16-Oct-2020	2	21-May-2021	17-Jan-2022
204	AR019	Baptist Memorial Hospital and Fowler Family Cancer Center - Jonesboro	04-Dec-2020	2	08-Jul-2021	03-Sep-2021
205	AR006	University of Arkansas for Medical Sciences	25-Sep-2019	3	10-Mar-2021	03-May-2022

Site Accrual Summary

#	Site	Site Name	Site Initial Approval Date	Accrual Total	Date of First Accrual	Date of Last Accrual
206	AL002	University of Alabama at Birmingham Cancer Center	03-Mar-2020	1	24-Feb-2021	24-Feb-2021
207	AK013	Fairbanks Memorial Hospital	10-Jun-2020	1	24-Jun-2020	24-Jun-2020
208	42086	Ehime University Hospital	11-Jan-2022	1	28-Apr-2022	28-Apr-2022
209	42064	Niigata University Medical and Dental Hospital	30-Nov-2021	2	13-Mar-2022	23-May-2022
210	42061	Saitama Medical University International Medical Center	03-Jun-2021	5	14-Jun-2021	29-Mar-2022
211	42036	Kyorin University Hospital	11-Jan-2022	1	31-Jan-2022	31-Jan-2022
212	42035	Hiroshima University Hospital	27-Oct-2021	2	12-Nov-2021	28-Jan-2022
213	42003	Gunma University Hospital	01-Feb-2022	1	21-Apr-2022	21-Apr-2022
214	42001	Tohoku University School of Medicine	18-Oct-2021	2	03-Mar-2022	16-Jun-2022
